# Suppression of *har-1/CHCHD10* phenotypes for ALS-FTD therapy discovery

**DOI:** 10.17912/micropub.biology.001598

**Published:** 2025-05-15

**Authors:** Audrey Labarre, Ericka Guitard, Gilles Tossing, J Alex Parker

**Affiliations:** 1 Centre de recherche du centre hospitalier de l'Université de Montréal (CRCHUM); 2 Neurosciences, Université de Montréal, Montréal, Quebec, Canada

## Abstract

Mutations in *CHCHD10* are linked to a variety of neurodegenerative diseases, including amyotrophic lateral sclerosis and frontotemporal dementia (ALS-FTD). The *Caenorhabditis elegans* orthologue of *CHCHD10* is *har-1 ,* and we investigated whether *har-1* mutants could be used for therapeutic discovery in ALS-FTD. Our results show that the small molecule pioglitazone and the probiotic *Lacticaseibacillus rhamnosus* HA-114 can alleviate *har-1* mutant phenotypes. These findings suggest that *har-1* mutants are suitable for modifier screens and could be adapted for high-throughput drug screening and microbiome studies to aid in discovering therapies for ALS-FTD.

**Figure 1. Suppression of mutant har-1 phenotypes through small-molecule or probiotic treatments:**
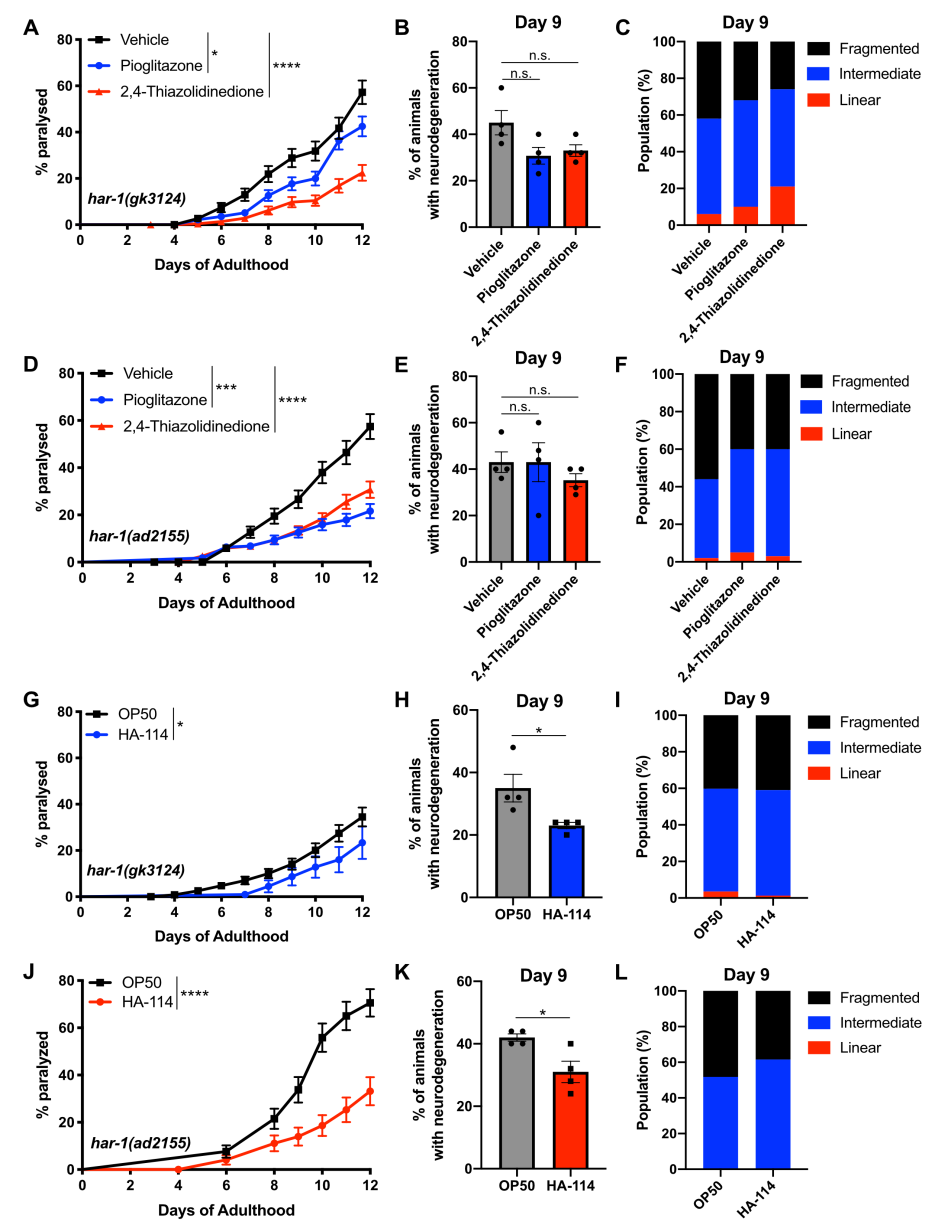
Pioglitazone and 2,4-thiazolidinedione suppressed ( **A** ) paralysis in *har-1 ( gk3124 )* mutants but failed to rescue ( **B** ) motor neuron degeneration on day 9 of adulthood. ( **C** ) Both compounds partially improved mitochondrial morphology defects (Linear: Vehicle vs. pioglitazone n.s., Vehicle vs. 2,4-thiazolidinedione *** *P* <0.001; Intermediate: Vehicle vs. pioglitazone n.s., Vehicle vs. 2,4-thiazolidinedione n.s., Fragmented: Vehicle vs. pioglitazone * *P* <0.05, Vehicle vs. 2,4-thiazolidinedione *** *P* <0.001). Pioglitazone and 2,4-thiazolidinedione suppressed ( **D** ) paralysis in *har-1 ( ad2155 )* mutants but had no effect on ( **E** ) motor neuron degeneration. ( **F** ) A partial rescue of mitochondrial morphology was observed when *har-1 ( ad2155 )* mutants were treated with pioglitazone or 2,4-thiazolidinedione (Linear: Vehicle vs. Pioglitazone n.s., Vehicle vs. 2,4-thiazolidinedione n.s.; Intermediate: Vehicle vs. Pioglitazone * *P* <0.05, Vehicle vs. 2,4-thiazolidinedione ** *P* <0.01, Fragmented: Vehicle vs. pioglitazone ** *P* <0.01, Vehicle vs. 2,4-thiazolidinedione ** *P* <0.01). ( **G** ) *har-1 ( gk3124 )* worms fed with HA-114 showed reduced paralysis compared to mutants fed with OP50 . ( **H** ) HA-114 prevented age-dependent neuronal loss in *har-1 ( gk3124 )* mutants ( **I** ) but did not affect mitochondrial morphology (Linear: OP50 vs. HA-114 n.s., Intermediate: OP50 vs. HA-114 n.s., Fragmented: OP50 vs. HA-114 n.s.). HA-114 rescues ( **J** ) paralysis and ( **K** ) neurodegeneration in *har-1 ( ad2155 )* mutants, while ( **L** ) partially restores mitochondrial health (Linear: OP50 vs. HA-114 n.s., Intermediate: OP50 vs. HA-114 *: p< 0.05, Fragmented: OP50 vs. HA-114 *: P< 0.05). *n.s.* : non-significant, * *P* < 0.05, *** *P* < 0.001, **** *P* < 0.0001.

## Description

*har-1* mutations lead to impaired motility and abnormal mitochondrial function in *C. elegans* (Woo et al., 2017). The robustness of these phenotypes suggests that they may be suitable for modifier screening. We have previously used *C. elegans* as a screening tool to identify small molecules that suppress paralysis and neurodegeneration phenotypes in multiple disease models, including ALS (Doyle et al., 2021; Fardghassemi et al., 2021; Patten et al., 2017; Schmeisser et al., 2017). However, few compounds emerged as specific modulators of mitochondrial genes or pathways. Recently, pioglitazone (PGZ), an oral antidiabetic drug from the thiazolidinedione family, was identified as a compound specific to disorders characterized by mitochondrial impairment, with the ability to reduce oxidative stress and improve mitochondrial bioenergetics (Mollo et al., 2019; Paciello et al., 2018; Yonutas et al., 2020). Interestingly, pioglitazone has also been recognized as a potential therapeutic for ALS in various models, including Drosophila and mice, and has been the subject of a clinical trial with promising results (Joardar et al., 2015; Schütz et al., 2005). Similarly, 2,4-thiazolidinedione (TZD), the core member of the thiazolidinedione family and the one with the simplest chemical structure, has been identified as a mitochondrial modulator with neuroprotective effects, particularly in Parkinson's disease (Wang et al., 2017).

Therefore, we tested PGZ and TZD in *har-1 ( gk3124 )* and *har-1 ( ad2155 )* worms to determine whether these compounds had protective effects. We observed that TZD treatment significantly reduced the paralysis phenotype in *har-1 ( gk3124 )* , while PGZ had a more modest impact on the motility phenotype ( **Fig. 1A** ). However, the reduction in motility defects was not accompanied by a significant decrease in GABAergic neurodegeneration ( **Fig. 1B** ). PGZ partially helped restore mitochondrial health by decreasing the number of animals with “fragmented” morphology ( **Fig. 1C** ). TZD had a more pronounced effect, as treated *har-1 ( gk3124 ) exhibit* ed a substantial increase in animals with “linear” mitochondria and a decrease in those scored as “fragmented” by day 9 of adulthood ( **Fig. 1C** ). Both PGZ and TZD treatment reduced paralysis in *har-1 ( ad2155 )* mutants ( **Fig. 1D** ), but they were ineffective at reducing motor neuron degeneration ( **Fig. 1E** ). Nonetheless, PGZ and TZD improved mitochondrial health in *har-1 ( ad2155 )* animals by significantly decreasing the numbers of both “intermediate” and “fragmented” scored animals ( **Fig. 1F )** . These results suggest that *har-1 ( gk3124 )* and *har-1 ( ad2155 )* worms exhibit a variety of features associated with ALS that can be modulated by FDA-approved drugs like PGZ, making these models useful tools for screening therapeutics.

Beyond small-molecule interventions, interest in the microbiome and dietary supplementation has arisen as a potential means to modulate disease phenotypes. Several studies have identified dysbiosis in ALS models and patients (Blacher et al., 2019; Burberry et al., 2020; Figueroa-Romero et al., 2020; Wu et al., 2015). Bacterial metabolites, including short-chain fatty acids like butyrate, play a role in energetic balance through mitochondrial processes (Besten et al., 2013). Although the functions of CHCHD10 remain unclear, mutations in *har-1* /CHCHD10 may influence energy production by altering mitochondrial function. Furthermore, energy deficits have been consistently recognized in ALS and are widely acknowledged as an integral aspect of the disease (Vandoorne et al., 2018).

In a laboratory context, worms are often cultivated on Petri plates containing *E. coli* OP50 as their primary food source and the main component of their natural microbiome. To determine whether dietary probiotic interventions could modify *har-1* phenotypes, we tested the probiotic strain *Lacticaseibacillus rhamnosus* HA-114 (hereafter referred to as HA-114) on both *har-1 ( gk3124 )* and *har-1 ( ad2155 )* mutants. We previously identified this strain as neuroprotective in transgenic models of ALS and Huntington's disease and speculated whether this neuroprotection extended to our *har-1* models (Labarre et al., 2022). We found that HA-114 significantly rescued paralysis phenotypes ( **Fig. 1G, 1J** ) and motor neuron degeneration ( **Fig. 1H, K** ) in the *har-1* mutants compared to worms fed control OP50 bacteria. Interestingly, HA-114 treatment did not affect mitochondrial morphology in *har-1 ( gk3124 )* worms ( **Fig.1I** ). However, HA-114 treatment reduced the proportion of *har-1 ( ad2155 )* mutants with a fragmented mitochondrial network ( **Fig. 1L** ). These findings suggest that dietary probiotic interventions in nematodes can modify paralysis and neurodegenerative phenotypes, and that HA-114 effectively reverses these phenotypes in age-dependent models of neurodegeneration associated with mitochondrial dysfunction. Collectively, these data highlight the potential of *har-1* mutants for therapeutic screens and encourage further investigation. Combined approaches should perhaps be explored to fully suppress the range of phenotypes observed in *har-1* mutants.

## Methods

***C* . *elegans* maintenance and strains**

Standard culturing techniques for *C. elegans* were used (Stiernagle, 2006) . Worms were kept on NGM agar plates with *E. coli* OP50 at 20 °C. Strains from the *C. elegans* Genetics Center (University of Minnesota) included N2 , VC3169 *har-1 ( gk3124 )* , DA2155 ( *har-1 ( ad2155 )* ), CB307 ( *unc-47 ( e307 )* ), PS6192 ( *syIs243[myo-3p::TOM20::mRFP + unc-119 (+) + pBS Sk+]* ), and TU3311 ( *uls60[ unc-119 p::YFP + unc-119 p:: sid-1 ]* ). The IZ629 strain ( *ufsl34[P unc-47 p::mCherry]* ) was provided by Dr. Michael M. Francis (University of Massachusetts). All mutant strains underwent four rounds of outcrossing to N2 . Strains were verified by PCR or sequencing. Some experiments were conducted by dissolving pioglitazone (20 uM, Sigma-Aldrich) or 2,4-Thiazolidinedione (20 uM, Sigma-Aldrich) into the NGM plates.

**Solid media paralysis assay**

From days 1 to 12 of adulthood, 40 L4 worms were transferred daily to fresh NGM plates and monitored for paralysis. Worms were deemed dead if they didn't move their heads when tapped and showed no pharyngeal pumping. Paralysis was the inability to move when prodded. Each experiment was conducted three times in duplicate at 20 °C.

**Neurodegeneration assay**

On day 9 of adulthood, worms were selected for *in vivo* motor neuron imaging to assess neural processes for gaps. Animals were immobilized in 5 mM levamisole in M9 buffer on 2% agarose pads. Imaging was done with a Zeiss Axio Imager M2 microscope with a 20X objective and 1.5 Optovar, using AxioVs40 software. Over four studies, at least 100 worms were scored per condition.

**Mitochondrial morphology analysis**

The structure of body-wall mitochondria was analyzed using the PS6192 ( *Pmyo-3::TOM20::mRFP + unc-119 (+) + pBS SK+* ) strain, which expresses a red fluorescent protein targeted to the outer mitochondrial membrane. This strain was crossed with both *har-1* genotypes to assess mitochondrial health. Age-synchronized adults at day 9 were immobilized on 2% agarose pads with 5 mM levamisole. Imaging utilized a Zeiss Axio Imager M2 microscope with a 40X objective and 1.5 Optovar, using AxioVs40 4.8.2.0 software. Mitochondria were classified as linear, intermediate, or fragmented based on Lu et al. (2011) criteria. At least 100 worms were scored per condition across four independent experiments.

**Statistics**

Survival curves for paralysis, lifespan, and stress resistance were compared using the Log-rank test with 60–100 worms examined per condition at least three times. For the neurodegeneration assay, one-way ANOVA with Dunnett's multiple comparisons was used. Two-way ANOVA with Dunnett's was used for mitochondrial morphology. Data were expressed as mean ± S.E.M., except mitochondrial morphology data, where error bars were removed for clarity. All statistical evaluations were conducted with GraphPad Prism v8.

## Reagents

**Table:** 

**Strain**	**Genotype**	**Available from**
N2	Wild type *C. elegans* strain	CGC
VC3169	*har-1 ( gk3124 ) *	CGC
DA2155	*har-1 ( ad2155 ) *	CGC
CB307	*unc-47 ( e307 ) *	CGC
PS6192	*syIs243* * [myo-3p::TOM20::mRFP + unc-119 (+) + pBS Sk+] *	CGC
IZ629	* ufsl34[P unc-47 p::mCherry] *	Dr. Michael M. Francis (University of Massachusetts, Worcester, MA)
XQ574	*har-1 ( gk3124 ); unc-47 ::mCherry *	Parker Lab
XQ602	*har-1 ( gk3124 )* ; [myo-3p::TOM20::mRFP + unc-119 (+) + pBS Sk+]	Parker Lab
XQ645	*har-1 ( ad2155* ) ; [myo-3p::TOM20::mRFP + unc-119 (+) + pBS Sk+]	Parker Lab
XQ658	*har-1 ( ad2155 );* unc-47 p::mCherry	Parker Lab

**Table:** 

**Compound**	**Catalog number**	**Source**
Pioglitazone	CDS021593	Sigma-Aldrich
2,4-Thiazolidinedione	375004	Sigma-Aldrich

**Table:** 

**Bacterial strain**	**Source**
*Escherichia coli OP50*	CGC
*Lacticaseibacillus rhamnosus* HA-114	Lallemand Health Solutions
